# Self-motion perception in the elderly

**DOI:** 10.3389/fnhum.2014.00681

**Published:** 2014-09-15

**Authors:** Matthias Lich, Frank Bremmer

**Affiliations:** Department Neurophysics, Philipps-Universität MarburgMarburg, Germany

**Keywords:** heading, aging, neural network model, area MST, virtual reality

## Abstract

Self-motion through space generates a visual pattern called optic flow. It can be used to determine one's direction of self-motion (heading). Previous studies have already shown that this perceptual ability, which is of critical importance during everyday life, changes with age. In most of these studies subjects were asked to judge whether they appeared to be heading to the left or right of a target. Thresholds were found to increase continuously with age. In our current study, we were interested in absolute rather than relative heading judgments and in the question about a potential neural correlate of an age-related deterioration of heading perception. Two groups, older test subjects and younger controls, were shown optic flow stimuli in a virtual-reality setup. Visual stimuli simulated self-motion through a 3-D cloud of dots and subjects had to indicate their perceived heading direction after each trial. In different subsets of experiments we varied individually relevant stimulus parameters: presentation time, number of dots in the display, stereoscopic vs. non-stereoscopic stimulation, and motion coherence. We found decrements in heading performance with age for each stimulus parameter. In a final step we aimed to determine a putative neural basis of this behavioral decline. To this end we modified a neural network model which previously has proven to be capable of reproduce and predict certain aspects of heading perception. We show that the observed data can be modeled by implementing an age related neuronal cell loss in this neural network. We conclude that a continuous decline of certain aspects of motion perception, among them heading, might be based on an age-related progressive loss of groups of neurons being activated by visual motion.

## Introduction

Visual navigation is of ultimate importance for our everyday life. Self-motion induces a movement of the image of the outside world on our retina. Gibson (1950) was the first to point out that this “Optic Flow” might be used to determine one's direction of self-motion (heading). A large body of studies (psychophysical, neurophysiological, and computational) was triggered by this seminal work.

Psychophysical studies have shown that human observers are indeed able to perceive heading in artificial as well as in pseudo-realistic displays (for review see e.g., Lappe et al., 1999; Britten, 2008). In such cases, the subjects' performance critically depends on the exact experimental conditions: 2-D vs. 3-D layout of the scene (van den Berg and Brenner, 1994; Palmisano, 1996; Beusmans, 1998; Grigo and Lappe, 1998; Lappe et al., 1999), stimulus duration (te Pas et al., 1998), number of flow field vectors (Warren et al., 1988) and fixation vs. real or simulated eye movements (Royden et al., 1992).

In general, observers can determine their heading direction during translational self-motion through an expanding radial flow field with an accuracy of up to 1–2° (Warren et al., 1988). Humans can also modify their walking speed depending on optic flow (Prokop et al., 1997). Furthermore, optic flow can be used for collision detection (Lee, 1980) and for the estimation of traveled distance (Bremmer and Lappe, 1999; Frenz et al., 2003; von Hopffgarten and Bremmer, 2010).

Given that many visual capabilities are learnt during early childhood (e.g., Ellemberg et al., 1999) it can be hypothesized that this applies also to self-motion perception. Yet, visual capabilities do not change only during early childhood. Previous studies showed that also in the elderly some decline occurs in various aspects of visual perception (for reviews, see e.g., Owsley, 2011; Andersen, 2012). In particular, visual functions such as visual acuity (Carter, 1982), contrast sensitivity (Owsley et al., 1983) and spatio-temporal integration (Sloane et al., 1988; Tulunay-Keesey et al., 1988; Elliott et al., 1990; Mestre et al., 1990) are known to deteriorate with age. Motion processing also declines with normal aging (Ball and Sekuler, 1986; Gilmore et al., 1992; Habak and Faubert, 2000; Baugh and Marotta, 2009; Callisaya et al., 2009; Roudaia et al., 2010). As an example, Bennett et al. (2007) showed, that older adults (>70 years) were less sensitive to motion, and were significantly less accurate at identifying the direction of 2-D-random dot cinematograms. Furthermore age related degrements in detecting collision events were observed (Anderson and Enriquez, 2006).

Also a decline of self-motion perception has been documented (Warren et al., 1989; Atchley and Andersen, 1998; O'Brien et al., 2001; Falkenberg and Bex, 2007; Chou et al., 2009; Allen et al., 2010; Kavcic et al., 2011). In most of these studies, a relative judgment of heading was investigated as a function of age. As an example, Warren et al. (1989) asked whether a simulated self-motion was to the left or right with respect to a stationary target. These authors found an age-related increase of threshold of about 1 degree, for both, translational and curvilinear heading, respectively. Roditi and Crane (2012) reported an age-related increase of thresholds for purely vestibular driven self-motion perception in the horizontal plane. While Duffy (2009) could not find an age-related decline for pure visual self-motion perception when only forward-heading was presented, he confirmed an increase of threshold when inward (simulated backward motion) and outward (simulated forward motion) were presented interleaved. Only few studies have tested absolute heading i.e., accuracy and precision, mostly in younger adults (d'Avossa and Kersten, 1996; Telford and Howard, 1996), some of them by presenting visual, vestibular, and/or combined stimulation (horizontal plane: Cuturi and Macneilage, 2013. Vertical plane, in a mixed-age cohort of subjects: Crane, 2014). Some of these studies reported systematic, but small undershoots of perceived heading (e.g., d'Avossa and Kersten, 1996), while others reported a perceptual overshoot (e.g., Cuturi and Macneilage, 2013). Even fewer studies have tested heading perception across age, either purely visual (Mapstone et al., 2006) or in a combined visual and vestibular approach (Crane, 2012). While Crane (2012) analyzed data with respect to the sensory modality (visual, vestibular, and spoken) but not age, Mapstone and colleagues presented four subject cohorts (young adults, middle-aged adults, older adults, and Alzheimer's disease patients) visual stimuli mimicking self-motion through a cloud of dots, in front of semi-realistic objects or through an environment with dots and objects. Subjects were asked to point toward the simulated direction of self-motion by means of a steering wheel (Mapstone et al., 2006). Remarkably, performance between subject groups did not differ for self-motion through a cloud of dots. Instead, subjects from all cohorts underestimated heading for greater eccentricities. Older subjects and patients performed worse, however, if only object motion was presented. Along the same vein, Billino and colleagues showed that various forms of motion perception deteriorate differently with age (Billino et al., 2008).

When considering self-motion perception and a possible decline in behavioral performance, one has also to consider the neurophysiological basis of self-motion perception and its age-related behavioral modulation. Many studies over the last three decades have provided clear evidence that visual cortical areas in the macaque brain are valid models for the processing of visual information in the human brain. Evidence from single-cell recording experiments in the macaque monkey suggests that two areas in the parietal cortex are critically involved in the neuronal processing of optic flow: the medial superior temporal area (MST) and the ventral intraparietal area (VIP). Both cortical regions have the adequate functional properties to extract heading information from visual self-motion stimuli (Duffy and Wurtz, 1991; Lappe et al., 1996; Bremmer et al., 2002a,b, 2010; Gu et al., 2006, 2007, 2008, 2010, 2012; Chen et al., 2011a,b). Perhaps even more important, modulation of neural activity in these areas by means of electrical microstimulation or reversible inactivation modulates monkeys' heading perception (Britten and van Wezel, 1998; Zhang and Britten, 2011). Functionally equivalent regions of macaque areas MST and VIP have been identified in the human temporal and parietal cortex (Bremmer et al., 2001; Wall and Smith, 2008).

The investigation of the processing of self-motion information has been very successfully complemented by theoretical studies over the years. Some of these studies focused on non-primate vision (e.g., Hennig et al., 2011; Hennig and Egelhaaf, 2012), while a number of groups developed artificial neural networks aiming at modeling human perception (Lappe and Rauschecker, 1993, 1994; Perrone and Stone, 1994; Beintema and Van den Berg, 1998; Grossberg et al., 1999; Beardsley et al., 2003; Fukushima, 2008; Browning et al., 2009; Gu et al., 2010; Saunders and Niehorster, 2010; Hennig et al., 2011; Hennig and Egelhaaf, 2012; Layton and Browning, 2014). Typically, network building blocks were modeled after neuronal elements, i.e., neurons in macaque cortical areas MT and/or MST. Most of these theoretical studies focused on reproducing previously observed neurophysiological response properties or human behavior, which is important in itself because it helps to better understand the functional role of certain building blocks like areas MT and MST. A smaller number of studies aimed not only at reproducing neuronal or behavioral response features, but also at making predictions for these features which could be tested in electrophysiological and behavioral follow-up experiments. Two such prototypical examples were introduced by Lappe and Rauschecker (1993, 1994) and, more recently, by Gu et al. (2010). Lappe and Rauschecker developed a two-stage model of visual self-motion processing. Importantly, functional properties of the two layers of this model were built after those in areas MT (input layer) and MST (output layer). Response characteristics resulting as predictions from this model were identified, among others, in neurophysiological follow-up studies in macaque area MST (Lappe et al., 1996; Lappe and Duffy, 1999; Bremmer et al., 2010). Gu et al. (2010) focused on the response properties of monkey MSTd neurons, assuming an adequate preprocessing of visual information up to this cortical stage. Different from the approach by Lappe and Rauschecker, their model incorporated e.g., also noisy responses. By applying various decoding regimes (Fisher information, maximum likelihood estimation, and population vector decoding), Gu and colleagues predicted systematic heading errors that were confirmed in follow-up studies in humans (Crane, 2012; Cuturi and Macneilage, 2013) as well as in the same study in monkeys (Gu et al., 2010). The neural basis of age-related changes in motion perception is as yet unclear. A decline in heading performance, if existent, could be related to a systematic change of the neural processing in areas MST and VIP. One such change is a progressive, age related cell death (Pakkenberg and Gunderson, 1997). While such changes are not directly accessible in humans, they can be modeled in artificial neural networks trained to determine heading.

Given the above mentioned findings, the goals of our current study were two-fold. In a first step, we aimed to functionally characterize in greater detail absolute heading judgments in the elderly as compared to younger controls. More specifically, we wanted to determine, if a certain parameter of our visual stimuli (presentation time, density of the visual stimulus, stereoscopic stimulation, and motion coherence) is of critical importance for an age-related decline of heading perception or for constant performance across age. If this was the case, it might point toward a putative neural basis and of an age related functional decline or, as an alternative, for constant performance across age. If, on the other hand, an age-related decline of heading perception would occur for all visual parameters under study, it would provide evidence for a general, non-specific decline in the neural processing of self-motion perception in the elderly. The second step of our study was related to this latter issue. Given a general decline of heading perception with age, as found in the experimental part of our study, we aimed to determine the consequences of an age related loss of neural tissue on heading perception, as mimicked by an artificial neural network. Based on our experimental data, the implemented cell loss did not affect the processing of certain visual parameters like disparity of the visual stimulus, but rather was affecting non-specifically the artificial neural network at use.

## Materials and methods

### Setup and subjects

Psychophysical experiments in normal human subjects were performed in visual virtual reality. Subjects sat comfortably on a chair while wearing an NVISOR-SX-head-mounted-display (HMD). This HMD was equipped with two reflective LCOS displays (1280 × 1024 pixels each at 60 Hz) with an effective resolution of 2.0 arc min and a monocular diagonal field-of-view of 60°.

The dots of the optic flow field were white (luminance 120 cd/m^2^) on a black background (<10 mcd/m^2^). They were moving at a mean velocity of 2.7° s (resulting from a simulated self-motion of 1.0 m/s) and had sizes between 0.2 and 10°. No boundary or pixel grid was visible. The distance between the eyes and the LC-display was 23 mm. The optic flow stimulus was generated by a custom program using Visual C++ (Microsoft, Redmond) in combination with OpenGL. All images were presented via an Nvidia-Quadro-Dualhead graphics card. For the projection of the scene, a non-symmetric camera frustum was applied for all experiments (23 mm–20 m). The viewing camera remained fixed und a modeling transformation was used to translate and orient the dots. Camera was specified by position, viewing direction, eye separation, distance to zero parallax and the near and far cutting planes.

Subjects constituted two experimental groups. One test group of older subjects (*n* = 10, mean age 67.8 years, *SD* = 5.9, min 58 years, max 80 years, 7 male, 3 female) and one control group of young adults (*n* = 10, mean age 26.2 years, *SD* = 4.7, min 22 years, max 35 years, 7 male, 3 female). Most participants were recruited students and employees from the University of Marburg, some were community volunteers. As a recruitment criterion, subjects had to report self-motion sensation. More specifically, subjects had to report illusory self-motion (vection), while the visual optic flow stimulus was presented in a test-sequence. In addition it was required for all subjects that they did not have any neurological or neuropsychological history. All older subjects were active car drivers and did not report any general handicap in everyday life. Subjects had normal or corrected-to-normal vision and gave informed written consent. All subjects were naïve as to the purpose of the study. Before the commencement of the study we tested and evaluated the stereoscopic depth perception with a random-dot stereotest (“Randotstereotest,” 400–20 s of arc at 16 in). The participants were required to have a stereo acuity of 40 s of arc. The procedures used in this study conformed to the Declaration of Helsinki.

### Experiment 1

**Figure 1:**
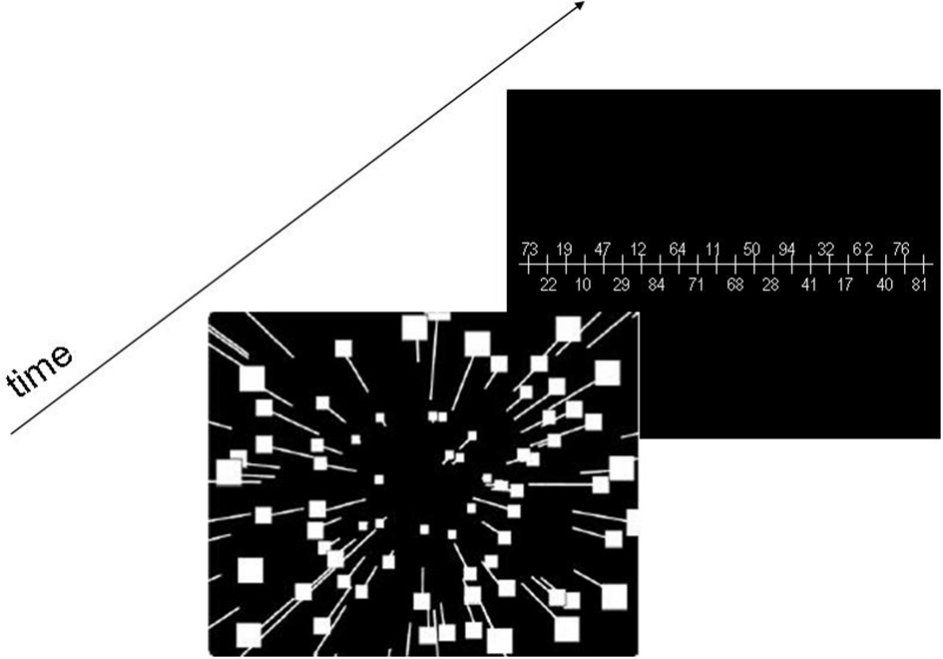
**Schematic Diagram of the stimulus design**. The presentation time of the optic flow stimulus varied between 200 and 2000 ms. After stimulus presentation a ruler with pseudo-randomized numbers was presented in the visual field and subjects were asked to indicate their perceived self-motion direction by giving the number on this ruler being closest to this perceived heading.

In a first step we examined the case of translational self-motion through a 3-D-cloud of random dots with fixed gaze. In such case, the focus of expansion indicates the current heading direction (Gibson, 1950). In this experiment identical stimulus sequences were presented to each eye, such that there was no horizontal disparity cue. The dots were randomly placed within the given camera frustum and moved radially outward from the focus of expansion (FOE). Simulated observer speed was constant (1.0 m/s), while presentation time varied between 200 and 2000 ms (200, 500, 1000, and 2000 ms). Dot density was varied such that the number of visible dots in the display ranged from 2 to 100 (2, 10, 50, 100 dots). We employed the method of constant stimuli, i.e., we used 7 heading directions, −15°, −10°, −5°, 0°, 5°, +10°, +15°, with the FOE positioned at any given trial at one of these directions on the horizontal meridian. After stimulus presentation a ruler was presented in the visual field and subjects were asked to indicate their perceived heading direction by reporting the number on this ruler being nearest to this perceived heading (Figure 1) either by verbal report (test group) or by entering the number on a key-board (control group). The numbers were distributed in a new pseudorandom sequence after each trial. They were spaced by 1° and presented alternately above and below the ruler. The subjects were not given feedback about their performance. Prior to the experiment, subjects were given 14 practice trials with feedback to familiarize themselves with the task. Figure 1 shows schematically the experimental timeline for a single trial. Each subject was tested 35 times (seven heading directions, five repetitions each) for each possible parameter combination (presentation time and number of dots), resulting in a total of 4 × 4 × 35 = 560 trials per subject.

In this and in all following experiments, we did not require our subjects to fixate a target during the experimental trials. Fixation of a target while attending to a heading stimulus would have imposed an attentional load on the subjects. It has been shown that dual tasking gets impaired in cognitive aging (e.g., Vandenbossche et al., 2014). Accordingly, if we had presented a fixation target in our experiments our results potentially could have been confounded by this dual tasking. The HMD in use did not allow to monitor eye-movements, which could be considered a drawback. This is especially true, since eye-movements impose further challenges on the visual system during self-motion: retinal motion might result from the simulated self-motion *per se* or from the own eye-movements (see e.g., Lappe et al., 1999; Bremmer et al., 2010). Accordingly, possible differences in heading perception between test and control groups might be confounded by different eye movement patterns of the two subject groups. On the other hand many studies on heading perception have been performed without eye-movement recordings (e.g., Billino et al., 2008). Hence, our study design was developed in line with previous research, possible limitations will be considered explicitly in the Discussion Section.

### Experiment 2

In a second set of experiments we examined the role of stereoscopic visual information (horizontal disparity) on heading performance. Horizontal disparity was obtained by generation of two identical stimulus sequences, each presented to one eye with a slightly different (disparate) viewing angle. For the projection of the scene, we used a non-symmetric camera frustum to exclude vertical parallax. The projection was performed with the off-axis-method (parallel-axis-asymmetric-frustum-perspective-projection) (Hodges, 1992; Lipton, 1997; Carrozzo and Laquaniti, 1998). In this concept the view vectors for each camera remain parallel. The dots extended out to a simulated distance of 20 m from the observer and had a minimum distance of 23 mm. Like in Experiment 1, stimulus elements increased in size when approaching the observer. Each subject performed 4 × 4 × 35 = 560 trials.

### Experiment 3

Non-coherent motion may be present in measurements obtained in real environments due to independent object motion. Such non-coherent motion is known to have an impact on the discrimination of 2-D frontoparallel motion which is age-related (Billino et al., 2008). Hence, in a third experiment we presented self-motion stimuli composed of non-coherent visual motion. We were interested on the correlation of aging and stereoscopic presentation of non-coherent visual motion. Dots randomly disappeared from the screen and appeared at a new position with new movement vectors. A certain percentage of dots moved coherently thereby simulating forward motion while the rest moved in random directions. During each frame, the dots had a certain probability to become noise vectors (Newsome and Pare, 1988). The correlation factor in our experiment was 0.15. This equals to a signal-to-noise-ratio (the ratio between coherent points and non-coherent points) of 0.177. In other words, 85% of the points were noise vectors while only 15% moved coherently to the simulated self-motion direction. Stimuli were presented either with or without horizontal disparity. Consequently we tested two conditions: non-coherent motion with non-stereoscopic stimulation and non-coherent motion with horizontal disparity. Each subject performed 4 × 4 × 35 = 560 trials.

### Estimation of maximum heading error

In order to valuate heading performance, it is necessary to have a detailed knowledge of the maximum heading error to be expected from the experimental procedure. To evaluate this maximum error, we performed a Monte-Carlo-simulation with different constraints. In the first condition, the selectable heading values were chosen with no constraint at all, i.e., each value was chosen randomly with the same probability. In a second condition, artificial heading values were randomly distributed but mainly within a certain region centered on the screen (Gaussian probability profile *p* = *a* · exp{−(*x* − *x*0)^2^/2σ^2^}; σ = 2°).

### Control task

In a control tasks we tested whether or not subjects were at all able to indicate targets in the display correctly (which might had been a critical issue especially for the test group). To this end a stationary white square (1.0 × 1.0°) was presented stereoscopically on the horizontal meridian. The positions were identical to the heading positions in the main experiment (−5°, 10°, 5°, 0°, 5°, +10°, +15°). The squares were presented for 200, 500, 1000, 2000 ms. After stimulus presentation a ruler was shown in the visual field and subjects were asked to indicate the perceived position of the square by giving the number on this ruler being nearest to this position. Each subject performed 4 × 4 × 35 = 560 trials of this experiment.

### Network simulation

In a previous study, Lappe and Rauschecker (1993) had developed a biologically plausible neural network model of optic flow analysis in the primate brain. This model predicts many of the principal findings of self-motion perception in humans. It is based on computational elements that mimic neurons in cortical motion processing areas MT and MST (Lappe et al., 1996).

We employed the exact model of Lappe and Rauschecker (1993) in order to mimic the consequences of healthy aging, i.e., the progressive loss of neural tissue, on heading perception. In this model optical flow serves as input (MT layer) and the evaluated heading is computed in the output (MST) layer. The model is a two-layer algorithm that estimates the actual self-motion by matching the motion parameters of the observer to the measured flow field according to a least-square criterion. More specifically, the input layer, which constitutes the flow field input, consists of 1200 MT-like units. 300 sets of *n* = 4 neurons are distributed within 50° eccentricity at random locations. The four neurons within each set have identical RF positions but are tuned to different directions of motion. Direction tuning is modeled by a rectified cosine function with preferred directions being equally spaced. The second layer represents a population encoding of the translational direction of the movement of the observer. Each MST-like unit of this second layer receives input from 30 image locations. The population of layer-two units forms a 3-D grid with 20 × 20 populations encoding one degree of translation-space each, and 20 pairs of neurons in each population, summing-up to 400 × 40 = 16000 units in layer-two. The connections and strengths between layer-one and layer-two were set fixed before the network was presented with any stimuli. So, in essence, the network did not learn synaptic weights. Instead, weights were given based on the mathematical equations translating movement vectors in 3-D space into local motion vectors projected onto the image plane, i.e., layer-one (see Lappe and Rauschecker, 1993, for further details). Given a specific flow field as input it determines which of the possible set of heading directions most likely generated this input flow field. From all possible flow fields it is calculated the one that minimizes the mean-squared difference between the measured flow field and all possible flow fields constructed from any combination of observer motion.

The model was aimed to be biologically plausible. Hence, the functional properties of the building units were formed after known properties of neurons in macaque areas MT and MST. Remarkably, the model not only allowed to replicate optic flow responses known at that time (e.g., Saito et al., 1986; Duffy and Wurtz, 1991). It also allowed to derive predictions concerning response selectivity of neurons in macaque area MST for certain optic flow stimuli. Indeed, these predictions could be verified in neurophysiological studies (Lappe et al., 1996; Lappe and Duffy, 1999; Bremmer et al., 2010). We implemented the process of aging in this model by a deactivation of a random selection of neurons in the MST layer. Neuron-deactivation probabilities were in the range of 1–10% according to neuropsychological data which suggest a rate of cell death of about 1–2% per decade of life (Jäncke, 2004; Raz et al., 2005).

### Data analysis

As a first step toward a quantification of the subjects' performance we fitted linear regression functions to the perceived heading. Qualitatively, the slopes of these functions reflect the ability to detect the various headings. The intercepts represent the offset along the axis of perceived heading, i.e., a possible bias with respect to straight-ahead motion.

As a second step, we determined the accuracy and precision of the subjects' performance. Accuracy was reflected by the absolute value of the deviation of the perceived from the real heading direction for all heading directions. Heading error was given by the arithmetic mean of these deviations. Precision was determined (for each heading direction) as the standard error of the distribution of perceived headings. In each data plot (starting with **Figure 4**), the precision is given by the error bar of data points.

To specify this analysis, the calculation of a data point in **Figure 4A**, is described in the following: raw data for each data point consisted of a sample size *n* = 350 (seven heading directions, five repetitions, 10 subjects). For each raw data value the absolute value of the deviation of the perceived from the real heading direction was determined. From these n absolute values the arithmetic mean (heading error) and the standard error (error bar) was calculated.

## Results

### Experiment 1

**Figure 2:**
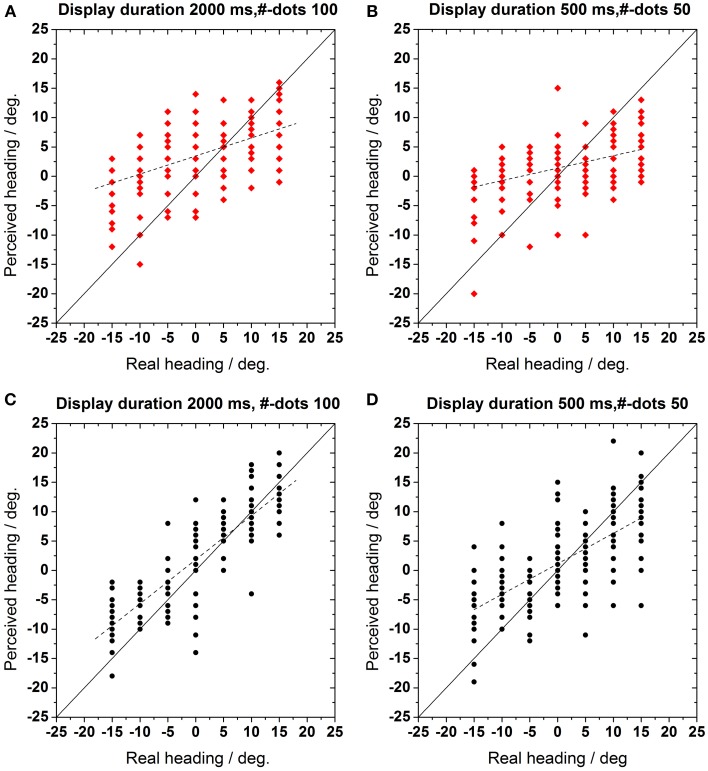
**(A,B)** Test group (*n* = 10 observer) and **(C,D)** control group (*n* = 10 observer): perceived heading direction as function of real motion direction. Here and in the following figures, red symbols show data from the test group, black symbols those from the control group. Each data point indicates a response in a single trial. Veridical perception is indicated by the solid identity line *f(x)* = *x*. The dotted lines represent a linear regression fit to the data. **(A,C)**: Display duration 2000 ms, number of dots 100, **(B,D)**: display duration 500 ms, number of dots 50.

In this experiment, we investigated, whether absolute heading was influenced by age similar as relative heading. (Figures 2A,B) (Test group data) and (Figures 2C,D) (Control group data) show a representative example for the perceived heading direction (ordinate) as a function of the real self-motion direction (abscissa). Veridical perception is indicated by the solid line *f(x)* = *x*. For the data shown, display duration was either 2000 ms with 100 visible dots in the display (left column, Figures 2A,C) or duration was 500 ms with 50 visible dots (right column, Figures 2B,D). As can be estimated from these two figures we observed a decline in the ability to perceive one's direction of self-motion for our test group: heading errors were much bigger for the test as compared to the control group, irrespective of the number of dots in the display. In order to quantify the subjects' performance we fitted linear regression functions to the data (*R*^2^ > 0.5 for all fits). The slope and the intercepts of these regressions were used for further analyses. Qualitatively, the slopes reflect the ability to perceive the various headings. Here, perfect behavior would correspond to a slope of 1.0. An inability to detect the various headings would be indicated by a regression function with a slope of 0.0. The slopes of the regression function were 0.22–0.51 for the test and the control group, respectively (500 ms, 50 dots). The intercepts represent the offset along the axis of perceived heading, i.e., a bias with respect to straight-ahead motion

**Figure 3:**
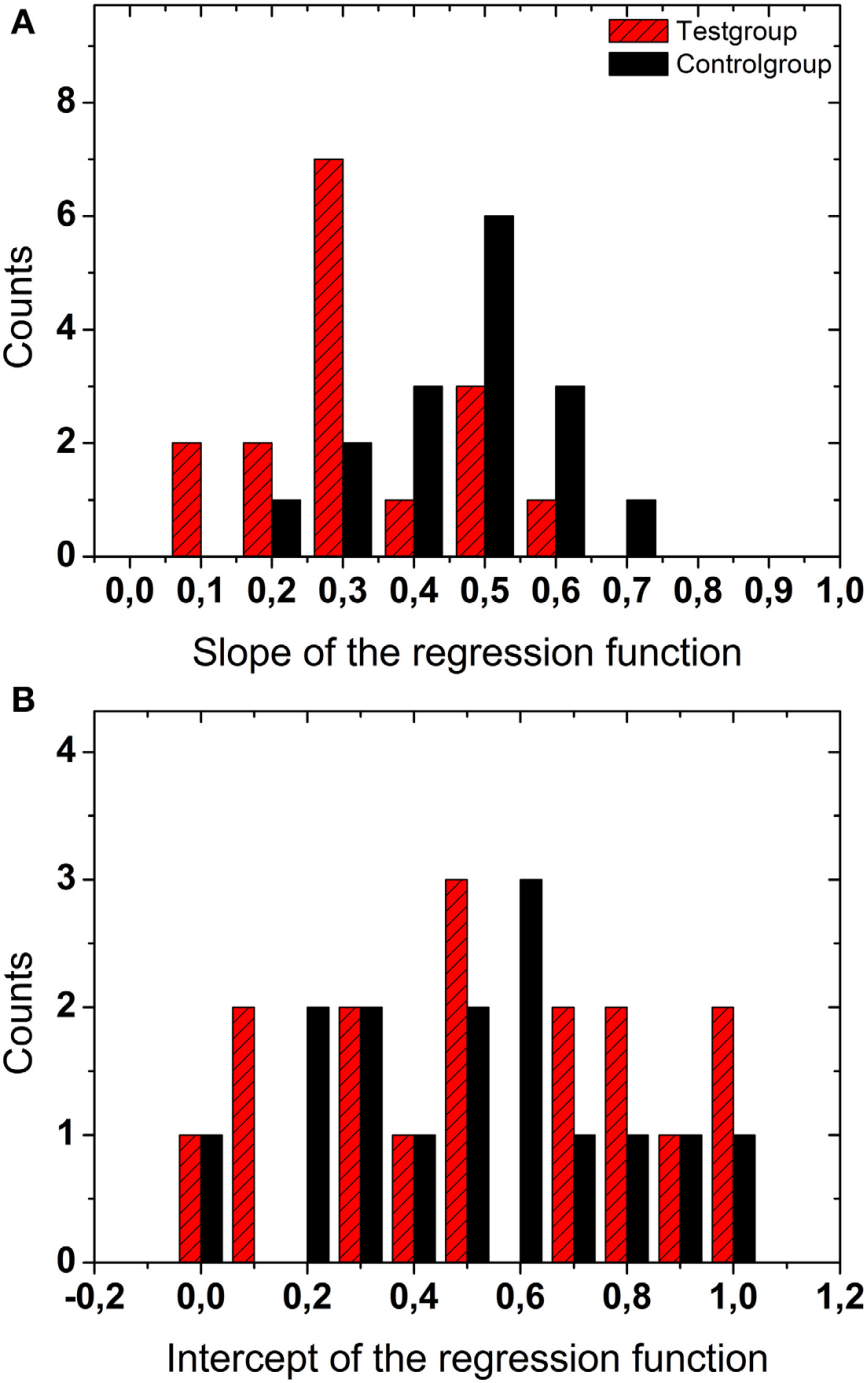
**Regression data**. The top panel **(A)** shows a histogram of the slopes of the linear regressions for all display durations and all numbers of dots for the test group (red hatched bars) and the control group (black bars). The bottom panel **(B)** shows the data for the intercepts of the regression functions.

The top panel in Figure 3 shows a histogram of the slopes of the linear regressions resulting from all display durations (200, 500, 1000, and 2000 ms) and all numbers of dots (2, 10, 50, 100), i.e., all 4 × 4 = 16 combinations for the test group (red hatched bars) and the control group (black bars). The bottom panel shows the data for the intercepts of the regression functions (same graphical scheme). The distributions of the slopes were significantly different for the test and the control group (*t*-test, *p* < 0.002, *df* = 15), i.e., they could be separated into two distinct groups. The slopes of the linear regressions from the test group were significantly smaller, i.e., more different from veridical, than for the control group. The distributions of the intercepts for the two groups were not statistically different (*t*-test, *p* > 0.4, *df* = 15).

**Figure 4:**
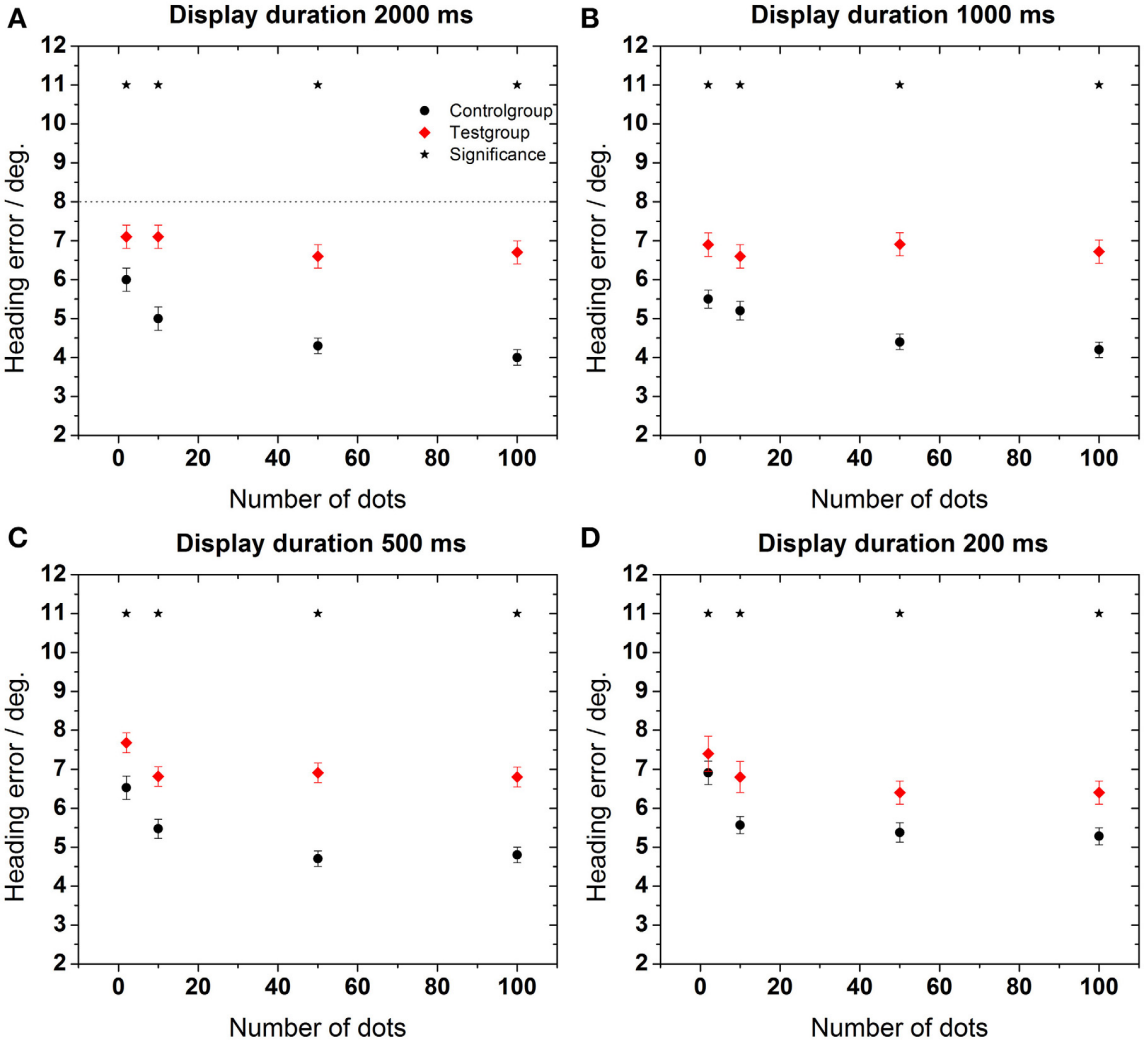
**Heading error as function of the number of dots in the display**. Data in each panel were obtained for a specific stimulus duration as indicated on top of each panel **(A–D)**. Error bars show standard error. Asterisks indicate significant differences between data from the control and the test group for a given number of dots in the display (*p* < 0.02, Mann–Whitney). The dashed line in the upper left panel represents an estimation of the maximum heading error as provided in the Section “Estimation of Maximum Heading Error.”

In a next step we aimed for a more detailed analysis of the heading errors. The data in Figure 4 show the heading error as function of the number of dots in the display for the four different presentation times. The heading error was calculated as described in the data analysis subsection.

As a general rule, the performance of the older subjects (test group) was worse than the performance of the younger subjects for all stimulus durations and all dot densities in the display [control group, Mann–Whitney, *p* < 0.02, followed by false discovery rate (FDR)-corrections (Benjamini and Hochberg, 1995)]. This difference was more pronounced for longer stimulus presentation times (≥500 ms). As an example, mean heading errors were 6.8 ± 0.3° for the test group and 4.7 ± 0.2° for the control group for a stimulus presentation of 2000 ms. In all cases, best performance occurred for longer presentation times for the control group. For this group, perceptual errors also decreased for higher point densities. Surprisingly, this was not the case for the test group, for which the error did not change significantly for a larger number of dots in the display for any stimulus duration (ANOVA, *p* > 0.7, *df* = 3, followed by Tukey corrected *post-hoc* tests).

**Figure 5:**
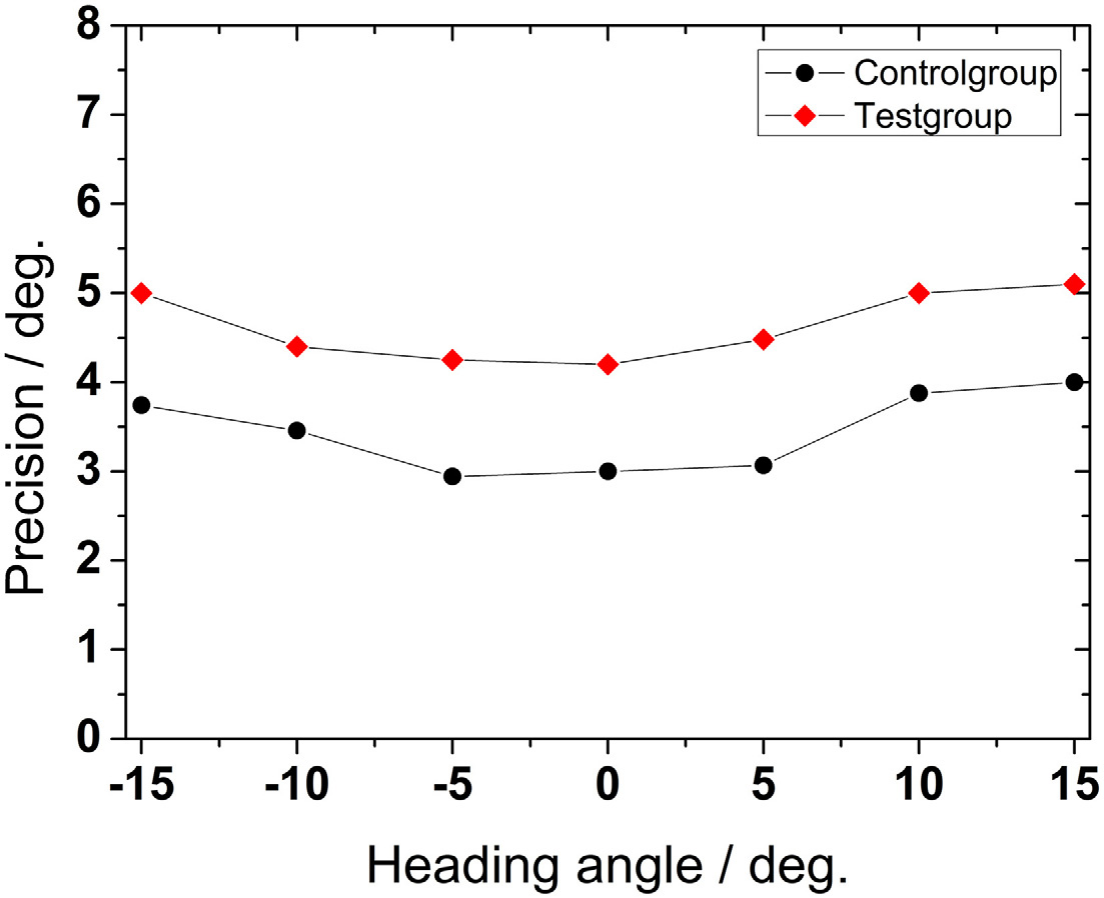
**Precision of heading detection**. Data show the standard deviation of the mean perceived heading directions as function of the presented heading direction, averaged across presentation time and number of dots for all subjects.

We also wanted to quantify the precision of the indicated heading direction. In Figure 5 we show the standard deviation (std) of the mean of perceived heading direction as a measure for precision for the test group and for the control group. Depicted std-values were obtained by averaging across all conditions (presentation times and number of dots) for all subjects and are shown as function of presented heading direction. Mean precision depended significantly on heading angle (ANOVA, *p* < 0.001, *df* = 6). Precision was higher, i.e., std-values were smaller for heading (close to) straight ahead (−5°, 0°, +5°). Precision decreased, i.e., std-values increased monotonically for more peripheral directions (−15°, −10°, +10°, +15°). For older subjects, precision was significantly worse than for the control group (Mann–Whitney, *p* < 0.001, followed by FDR-corrections).

### Experiment 2

**Figure 6:**
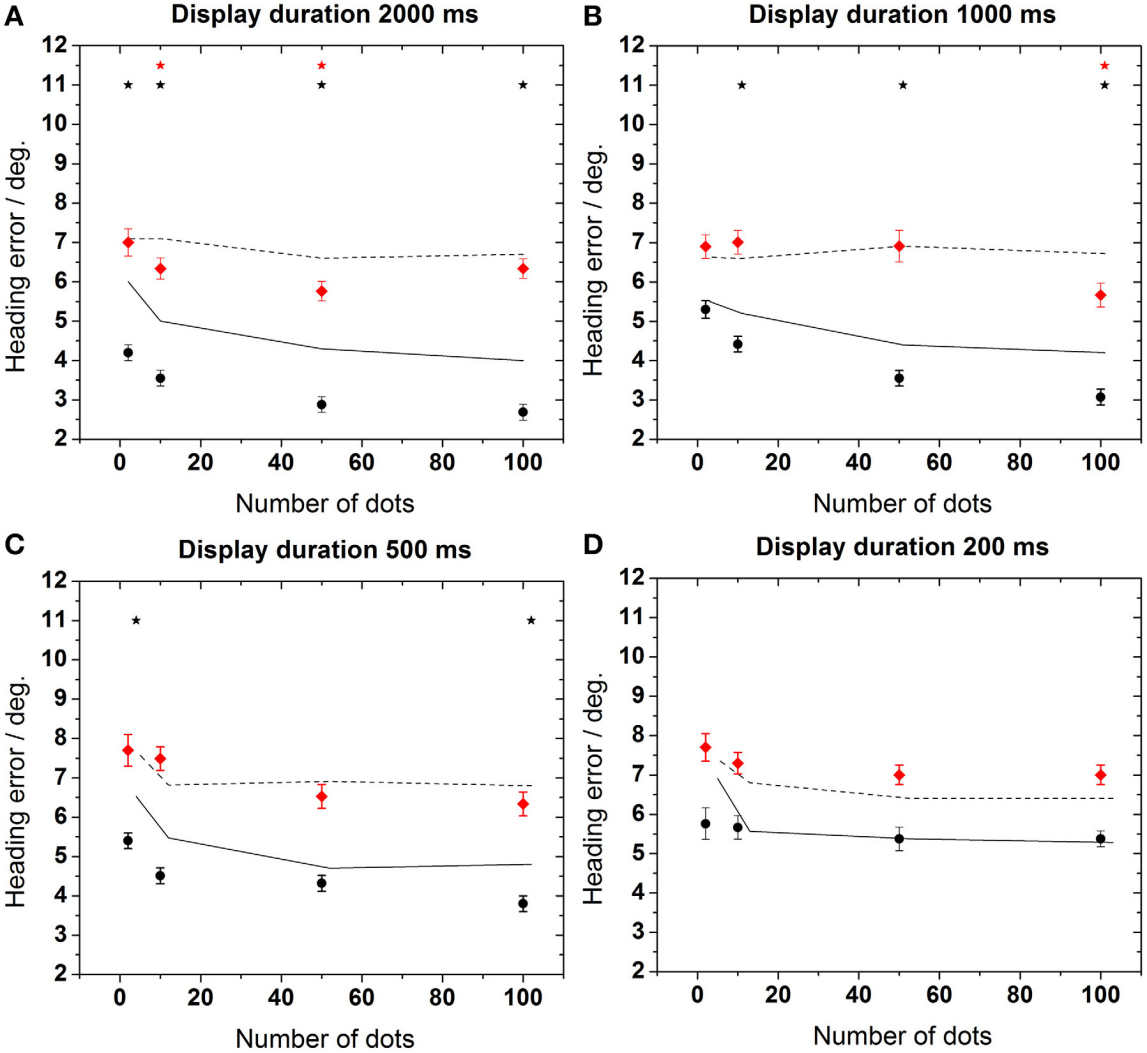
**Heading error as function of the number of dots during stereoscopic stimulation**. The solid lines (control group) and dashed lines (test group) depict the results of Figure 4 (non-stereoscopic stimulation) for comparison. Asterisks indicate significant differences between data from non-stereoscopic simulation and stereoscopic simulation of the control group.

In a second set of experiments, we examined the role of additional stereoscopic visual information on the subjects' heading performance (Figure 6). We tested, whether stereoscopic stimulation improved performance. For both groups, we observed an overall improvement of heading perception. We considered such an improvement to occur for a given presentation time if performance in the stereoscopic condition was significantly better than in the non-stereoscopic condition for at least 3 of the 4 possible dot densities. According to this criterion, an improvement was found in test and control groups for longer presentation times (≥1000 ms for the control group and 2000 ms for the test group) and a larger number of dots (Mann–Whitney, *p* < 0.001–*p* < 0.05, followed by FDR-corrections). In Figure 6, asterisks indicate these statistical differences between conditions (stereoscopic vs. non-stereoscopic stimulation). Best performance, i.e., the minimal error was 2.7 ± 0.2° for the control group (2000 ms, 100 dots) and 5.7 ± 0.3° for the test group (1000 ms, 100 dots). Like in Experiment 1, heading performance was generally worse for the test group as compared to the control group (Mann–Whitney, *p* < 0.001–*p* < 0.05). Likewise, subjects of the control group took advantage of the higher dot density: perceptual errors monotonically decreased for an increasing number of dots in the display.

**Figure 7:**
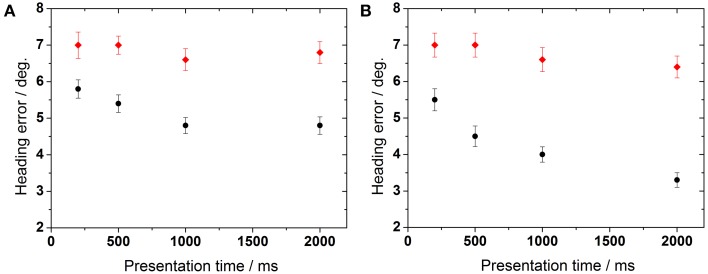
**Influence of presentation time on heading performance**. The left panel **(A)** represents data obtained during non-stereoscopic stimulation, right panel **(B)** shows data obtained during stereoscopic stimulation.

We also wanted to quantify the overall influence of stimulus presentation time on behavioral performance. To this end, we averaged heading error across all dot densities for monoscopic and stereoscopic presentation (Figure 7). For the monoscopic task (Experiment 1), control group's performance significantly improved with presentation time (ANOVA, *p* < 0.04, *df* = 3, followed by Tukey corrected *post-hoc* test). Older subjects' performance showed a tendency to improve (ANOVA, *p* > 0.053, *df* = 3, Tukey corrected). For the stereoscopic task (Experiment 2), heading perception in the control group improved with increasing presentation times (ANOVA, *p* < 0.02, *df* = 3, Tukey corrected). Again, older subjects showed only a non-significant trend toward improved heading perception with increasing presentation times (ANOVA, *p* > 0.05, *df* = 3, Tukey corrected).

### Experiment 3

**Figure 8:**
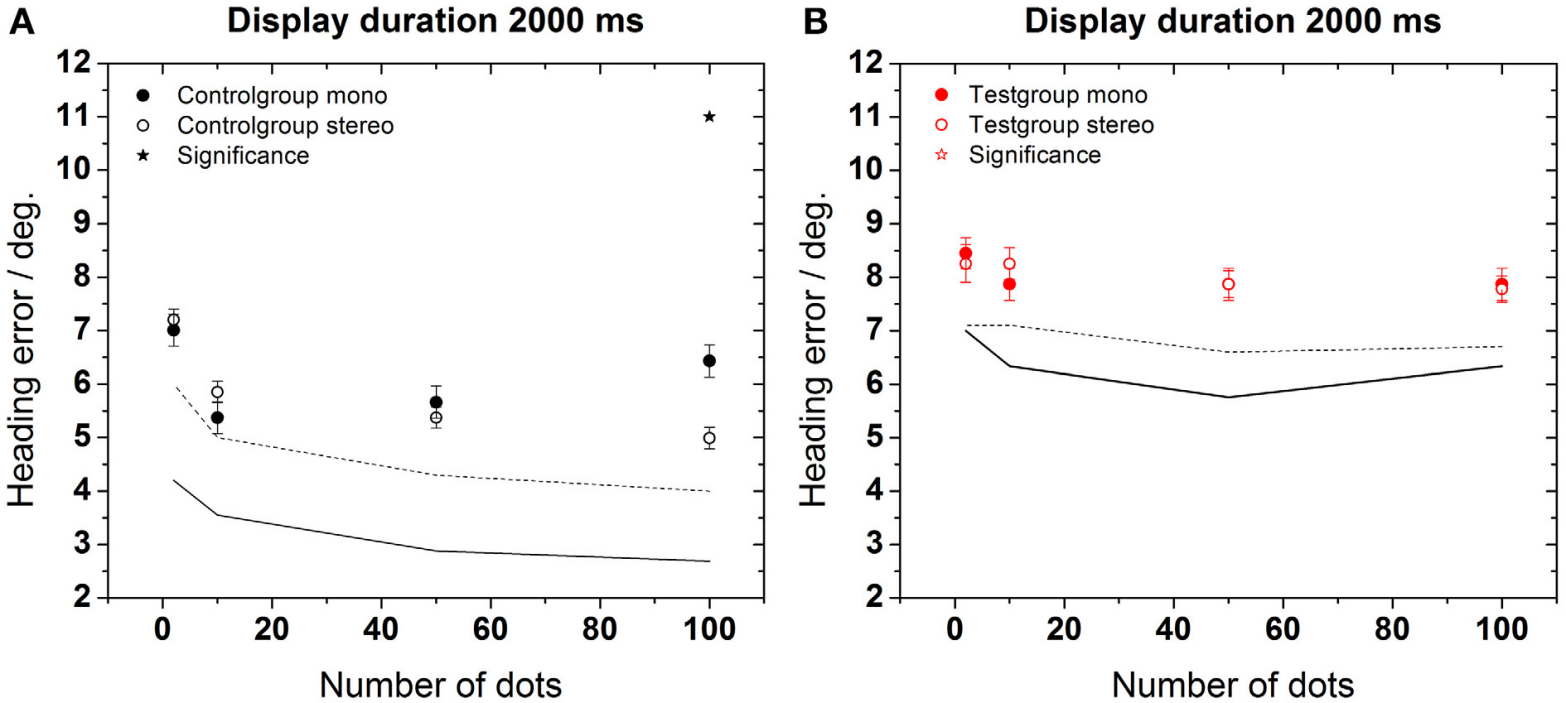
**Influence of non-coherent motion on translational heading**. The left panel **(A)** shows data from the control group for non-coherent motion with stereoscopic stimulation (open symbols) and without (filled symbols). Data from the test group are shown in an analog manner in the right panel **(B)**. Performance with and without stereoscopic stimulation was significantly different only in the control group for only the largest number of dots in the display as indicated by the asterisk. The dashed lines in each panel represent the results from Experiment 1 as shown in Figure 4 (coherent motion, non-stereoscopic stimulation). The solid lines in each panel represent the results from Experiment 2 as shown in Figure 6 (coherent motion, stereoscopic stimulation).

In a third set of experiments we investigated the influence of non-coherent motion on heading perception. In this case, only 15% of the dots moved according to a given heading direction, 85% of the dots moved non-coherently. We tested, whether the performance for coherent motion was better than for non-coherent motion for both groups. Figure 8 shows a representative result for a presentation time of 2000 ms. For comparison, the dashed line (data from non-stereoscopic stimulation) and the solid line (data from stereoscopic stimulation) represent the results shown already in Figures 4, 6, i.e., when subjects were presented with 100% coherent motion. As can be seen from Figure 8, partially coherent motion with horizontal disparity impaired performance in both, the test group and the control group, respectively. The test group (data shown in the right panel) was significantly impaired in the stereoscopic condition with an additional mean heading error of 1.6°compared to coherent motion resulting in an overall error of 8°, represented as the mean across all dot conditions (Mann–Whitney, *p* < 0.03, followed by fdr-correction). For the control group (data shown in the left panel) we observed an additional heading error of 2.6° for the stereoscopic condition resulting in a total mean error of 5.9° (Mann–Whitney, *p* < 0.03, followed by fdr-correction).

### Estimation of maximum heading error

The behavioral performance of the test group in some cases was rather poor. We therefore wanted to ensure that the performance was not biased by experimental artifacts or, even worse, due to an inability of the test subjects to solve the given task. We therefore, compared our experimental results with three different hypothetical performances.

**Figure 9:**
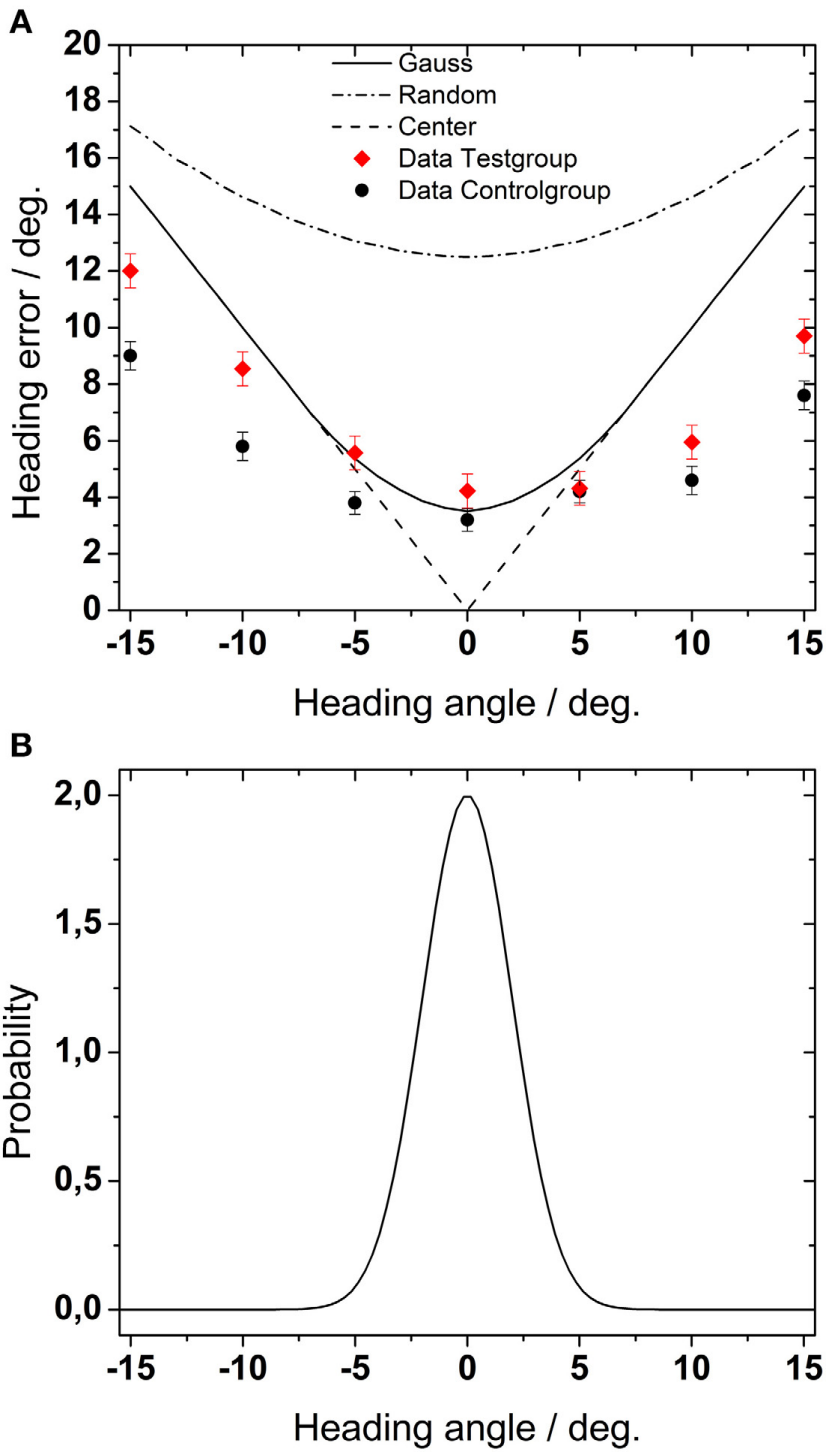
**(A)** Estimation of maximum heading error. The dashed-dotted line shows the heading error for purely random behavioral performance, i.e., if heading values were randomly chosen with the same probability. The solid line represents simulated heading error if heading values were chosen according to a Gaussian probability function centered on straight-ahead [as shown in **(B)**, see text for details]. Finally, the dashed line represents simulated heading error for choosing always the center of the screen.

In the first, we determined the maximum possible heading error for different behavioral settings (Figure 9). In a first approach, we modeled random behavioral performance, i.e., we assumed that heading values would have been chosen by our subjects randomly with the same probability. This theoretical performance would have resulted in a mean heading error of 14° as averaged across all heading directions. All experimental results from experiments 1,2, and 3 were well below this estimated random performance. Accordingly, we conclude that subjects did not chose perceived heading at random but rather solved the task as expected.

For a second simulation, we modeled again a random behavior. But now we assumed that subjects would have a bias for the central direction. This bias was mimicked by a Gaussian probability function centered on straight-ahead. Such a behavior would have led to a mean heading error of 8°, i.e., a value comparable with the maximum average error found in the most challenging parts of our experiments. The simulated error function had a minimum for the straight ahead direction and increased for more peripheral positions. In comparison to the performance drawn from a uniform probability distribution, the resulting errors were generally smaller.

Finally, in a third approach, we assumed that subjects would have always chosen straight-ahead as their perceived heading. This behavior would have resulted in a mean heading error of 8.6°.

Figure 9 shows the results for the different probability distributions. The symbols in Figure 9A depict the experimental data from Experiment 1 for the test group and for the control group, averaged across all conditions (presentation times and number of dots). Except for central heading, the average error in our experimental data as obtained from all simulated self-motion directions was in all cases smaller than any simulated error. Most critically, perceived heading was smaller than would have been expected if subjects had responded at random.

### Localization of stationary targets

The simulations described above clearly indicate that subjects reliably solved the task and did not perform at random. Yet, the observed errors for the test group as well as for the controls could have resulted from a general inability to interact in a virtual environment. In order to test for such an alternative explanation for the observed results, we tested subject's performance to localize stationary targets in the display. Behavioral performance did not differ significantly for both groups of subjects (See supplementary Figure 1. Mann–Whitney, *p* = 0.66): average error across all conditions (presentation times) was 1.7°. This result re-assures that subjects were generally able to indicate heading in the display. We therefore, conclude that characteristics like technical familiarity with the display and possibly existing age related changes in motor response did not interfere with behavioral performance in our heading task.

### Interim conclusion

Experimental data yielded the following results. First, our data revealed an influence of age on absolute heading performance. Second, we observed an overall improvement of absolute heading perception with stereoscopic stimuli. Third, we found that adding horizontal disparity to non-coherent translational flow field does not generally improve the absolute heading performance. Estimation of maximum heading demonstrated, that the average error in our experimental data was smaller than the simulated and the random generated error.

### Network simulation

Our data clearly revealed an age-related impairment in heading discrimination. The neural basis of such a decline in behavior is as yet unclear. Anatomical studies suggest an age related cell loss with a rate of cell death of about 1–2% per decade of life (Jäncke, 2004; Raz et al., 2005). To our knowledge, there is no evidence that cell loss is coupled to certain functional properties of neurons wihin certain areas or brain regions. Instead, cell loss appears to affect the entire population of individual areas or parts of the brain. We wanted to test the plausibility of a neuronal cell loss as basis for our findings. To this end we built on a neural network model developed by Lappe and Rauschecker (1993). A combined experimental and theoretical study had shown that neurons in this network behave similarly to neurons in primate motion sensitive areas MT and MST (Lappe et al., 1996). Accordingly, we implemented the process of aging in this model by a “deactivation” of neurons in the MST layer with a certain probability. We did not select network neurons with certain response features (e.g., receptive field location, direction preference, etc.). Instead, network neurons were selected at random. Based on published data (Jäncke, 2004) we simulated an age-related cell loss of 1–10%.

**Figure 10:**
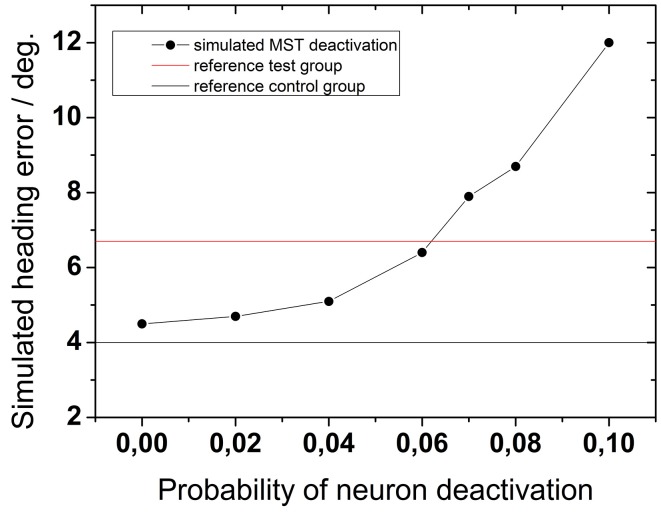
**Network simulation: the effect of aging on heading performance**. The solid horizontal lines represent as a reference the experimental results from both subject groups as obtained for 100 dots and 2000 ms (solid black line: test group; solid red line: control group). Black symbols represent simulated heading errors as function of different neuron-deactivation-probabilities.

Figure 10 shows the results of a network simulation assuming 100 dots in the display. For comparison the average experimental results for older and younger subjects are shown as black (control group) and red lines (test group). Qualitatively, the experimental data had the same magnitude as the expected theoretical values.

More importantly, however, the simulations demonstrate that an increasing probability of neuronal deactivation resulted in a larger simulated heading error. While a deactivation-probability of 2% yielded an average heading error of 4.7°, a deactivation-probability of 10% resulted in an average heading error of 12°. According to our network simulations, an experimental heading error in the range of 8° (as evident from our test group) resulted from a cell loss in the range of 6–8%, which in turn is the estimated cell loss of subjects in their 7-th decade of life.

## Discussion

We investigated the influence of aging on absolute heading perception. Our study had two major goals: firstly, we wanted to functionally characterize absolute heading perception in the elderly as compared to younger controls. Secondly, we wanted to determine on a theoretical basis, whether or not a progressive, non-specific loss of neural tissue, which accompanies healthy aging, would allow to explain impaired heading perception in the elderly.

As to the first goal, we quantified the influence of four different visual stimulus parameters, i.e., (i) number of dots in the display, (ii) presentation time, (iii) motion coherence, and (iv) stereoscopic stimulation, on absolute heading perception. Stimuli were presented to older test subjects and younger controls, who had to indicate perceived heading by means of a ruler. Heading accuracy for elderly subjects was generally worse than for younger observers. Surprisingly, elder subjects were impaired in their ability to make use of increasing visual information in the display as provided by longer (non-stereoscopic) display durations and a larger number of dots. In comparison, the performance of the test groups significantly increased with increasing visual information. From our control experiment with stationary targets we conclude, that the deteriorated performance of the older test groups was not caused by (i) age-related changes in sensori-motor responses and/or (ii) memory deficits. Our results are in line with findings from Warren et al. (1989) who tested relative heading perception, i.e., subjects had to indicate whether simulated self-motion was to the left or to the right of a stationary target. These authors found a small but statistically significant age related decline of about 1° in the ability to see where one is heading. Furthermore, Berard et al. (2009) showed that older adults were impaired to use optic flow cues to guide their locomotion. Our results differ, however, from those of Mapstone et al. (2006). In their study, subjects had to indicate by means of a steering wheel the perceived heading. The authors compared the behavioral performance of healthy younger, middle-aged and older subjects and patients suffering from Alzheimer's disease. When self-motion was simulated by a 3-D cloud of dots rather than moving objects, no differences in absolute heading perception was found. In all four test groups, heading perception was not veridical, but shifted toward straight-ahead. While also in our study subjects from both groups underestimated eccentric heading, errors in the test group, i.e., the elderly, were significantly larger than in young adults. The difference between our results and those of Mapstone and colleagues might be caused by the visual stimuli employed in the two studies. While in our study, stimuli mimicked translation through a 3D cloud of dots, those of Mapstone and colleagues simulated translation toward a vertical plane of dots. It is known from the literature that the spatial layout of a scene significantly influences performance in heading tasks (Lappe et al., 1999).

At first glance, our data also seem to be in contrast with data from a study by Billino et al. (2008). These authors found a rather stable heading performance across age while other types of motion perception (2-D linear motion and biological motion) were impaired in older subjects. Yet, the experimental paradigms in both studies concerning heading differed substantially. Billino and colleagues employed a relative heading task, i.e., a two alternative forced choice task with a fixed number of dots and a maximal heading angle of 5.6°. In our study, on the other hand, subjects had to indicate absolute heading. Furthermore, as can be seen in Figure 9, behavioral performance for straight-ahead direction, which was critical in the study of Billino and colleagues, was only slightly different for test and control subjects in our study.

Eye movements challenge visual perception (e.g., Bremmer and Krekelberg, 2003). This consideration is of critical importance for our results because the presentation of optic flow stimuli can elicit spontaneous eye movements (Lappe et al., 1999; Bremmer et al., 2010). In our study, we avoided to require our subjects to fixate a target during presentation of the self-motion stimuli. Fixation would have been a second task in addition to heading perception. Such a second task could have potentially confounded our data as it has been shown that dual tasking gets impaired in cognitive aging (e.g., Vandenbossche et al., 2014). The head-mounted display in use did not allow to monitor eye-movements. Accordingly, the observed differences in heading perception between test and control groups could have been confounded by different eye movement patterns of the two subject groups. Age-related differences in eye-movement behavior have indeed been demonstrated before. As an example, previous studies have shown that the latency of saccades and smooth pursuit increase with age (Munoz et al., 1998; Knox et al., 2005). On the other hand, there is no evidence that the accuracy of eye movements differs across age (Bennett et al., 2007). In addition, many studies on heading perception have been performed without eye-movement recordings (e.g., Billino et al., 2008). Accordingly, our experimental approach was a trade-off between minimal restrictions of the observers and possible confounds due to eye movements. Nevertheless, it was in line with previous research. Bearing these considerations in mind, our results suggest that an age-related modulation of heading perception might rather be associated with a modulation in motion processing, possibly due to changes in neural processing in specific cortical areas.

The second set of experiments in our study showed for the first time an overall improvement of heading perception with stereoscopic stimuli (horizontal disparity). This improvement was found for the test and the control group for increasing presentation times and numbers of dots. This result is consistent under the assumption that stereopsis needs up to 1000 ms to build up (Harwerth et al., 2003). The lack of an enhancing effect at brief presentation times could also be due to vergence eye movements. This is because studies have shown that the latency of accommodative vergence increases with age and that disparity-driven accommodation declines with age (Heron et al., 2001; Rambold et al., 2006). Yet, since we did not measure eye movements in our current study, we can only speculate that this issue contributed (at least in part) to our observed results.

In any case, our findings provide further evidence for the hypothesis, that the addition of stereoscopic motion cues directly and explicitly enhances self-motion perception. Some studies already had demonstrated an interaction of optic flow perception and stereoscopic depth vision. Lappe and colleagues suggested a disparity-dependent weighting function such that more distant flow vectors contribute more to the optic flow processing than near ones (Lappe and Grigo, 1999; Lappe et al., 1999). It had also been shown, that adding disparity to a simulated motion makes heading judgments more tolerant to noise (van den Berg and Brenner, 1994). There is also some evidence that perceived 3D-shape influences perceived heading (Beusmans, 1998) and that the addition of stereoscopic motion cues induces a more compelling vection (Palmisano, 1996).

These behavioral observations concerning stereoscopic stimulation in humans are paralleled by findings from neurophysiological studies in visual self-motion areas of the animal model, i.e., the macaque monkey. Observations from recordings in macaque area MST suggest also a contribution of depth to heading. Heading tuning improved in the majority of MST neurons when depth planes were included as compared to non-depth conditions (Upadhyay et al., 2000). In addition, Roy et al. (1992) described a disparity-dependent behavior for MST neurons. Our data confirm the enhancing effect of stereoscopic stimulation for heading perception.

Finally, the third set of experiments revealed that adding horizontal disparity to a non-coherent translational flow field does not generally result in a better heading performance. Instead, heading accuracy in the control group improved only for long presentation times (2000 ms) and a larger number of dots (*n* = 100). Only in this case, the additional motion-depth-cue (horizontal disparity) made the visual system more robust against noise.

Partially coherent motion with horizontal disparity impaired performance in both groups. This is in line with findings, that the accuracy of heading judgments is related to coherence of the dots presented in the display (Gu et al., 2008; Fetsch et al., 2009). Further, it was shown, that older subjects were impaired on a test of motion coherence patterns for stimuli of a slow to medium speed (Snowden and Kavanagh, 2006).

We were also interested in the question on how exactly aging is represented in the brain. To this end, we aimed to modify an already existing model on heading perception by means of a biologically plausible correlate of cognitive aging. Based on MRI-measures it was shown that some brain regions shrink with age (Raz et al., 2005) which at least in part is due to a neuronal cell loss (shrinking could also reflect a loss of glia or loss of blood vessels). Another hypothesis is a progressive drop of the functional properties of neurons. As an example, it has been shown that aging increased the level of noise and decreased directional tuning of neurons in the primary visual cortex of cats (Hua et al., 2006) and macaque monkeys (Leventhal et al., 2003; Yu et al., 2006). Our network simulation was based on the assumption of a global cell loss as proposed e.g., in Pakkenberg and Gunderson (1997). Given these different hypotheses concerning the neural correlate of a healthy aging brain, we decided to base our network simulation on the assumption of a global cell loss as proposed e.g., in Pakkenberg and Gunderson (1997). As neural network we selected the model introduced by Lappe and Rauschecker (1993). As mentioned already in the Introduction, a number of neural network models on the processing of self-motion information have been published over the last two decades. The ultimate goals of the different models are quite different. A recent model by Gu et al. (2010) allows to include specific features of a population of cells like independent or correlated noise. It also allows to process visual and vestibular information. Accordingly, this model is perfectly suited to investigate from a theoretical point of view heading, the interaction of different sensory information, response variance etc. The model by Gu and colleagues thereby focuses on information processing in macaque area MSTd. It assumes for good reason that visual information has been processed appropriately along the dorsal pathway up to area MT. Other models have focused on the processing of visual information, but have addressed also the information processing in macaque area MT. One such example was the model by Lappe and Rauschecker (1993), which has demonstrated its validity in a number of follow-up studies (Lappe et al., 1996; Lappe and Duffy, 1999; Bremmer et al., 2010). In this latter model we mimicked healthy aging by inactivating a certain proportion of randomly chosen neurons. According to the above mentioned work e.g., by Pakkenberg and Gunderson (1997) there is a general cell loss of approximately 1–2% per life decade starting at the age of 30. Hence, there would be a cell loss of approximately 10% in the 7-th decade of life. The resulting error in our simulation was in the same range as the one observed in our test group. Our results cannot verify or falsify the one or the other hypothesis concerning the neural basis of a healthy aging brain. Our results also cannot verify or falsify the one or the other network model of heading perception. Our results, however, demonstrate that combined experimental and theoretical approaches indeed allow to explain certain behavioral effects and to postulate new hypotheses (here: reduced heading performance due to a general neural cell loss) which can be critically tested in upcoming studies.

### Conflict of interest statement

The authors declare that the research was conducted in the absence of any commercial or financial relationships that could be construed as a potential conflict of interest.

## References

[B1] Allen H. A. Hutchinson C. V. Ledgeway T. Gayle P. (2010). The role of contrast sensitivity in global motion processing deficits in the elderly. J. Vis. 10, 15 10.1167/10.10.15 20884480

[B2] Andersen G. J. (2012). Aging and vision: changes in function and performance from optics to perception. Wiley Interdiscip. Rev. Cogn. Sci. 3, 403–410 10.1002/wcs.1167 22919436 PMC3424001

[B3a] Anderson G. J. Enriquez A. (2006). Aging and the detection of observer and moving object collisions. Psychol. Aging 21, 74–85 10.1037/0882-7974.21.1.74 16594793

[B4] Atchley P. Andersen G. (1998). The effect of age, retinal eccentricity, and speed on the detection of optic flow components. Psychol. Aging 13, 297–308 10.1037/0882-7974.13.2.297 9640589

[B5] Ball K. Sekuler R. (1986). Improving visual perception in older observers. J. Gerontol. 41, 176–182 10.1093/geronj/41.2.176 3950343

[B6] Baugh L. A. Marotta J. J. (2009). When what's left is right: visuomotor transformations in an aged population. PLoS ONE 4:e5484 10.1371/journal.pone.0005484 19436727 PMC2677156

[B7] Beardsley S. A. Ward R. L. Vaina L. M. (2003). A neural network model of spiral-planar motion tuning in MSTd. Vision Res. 43, 577–595 10.1016/S0042-6989(02)00608-9 12595004

[B8] Beintema J. A. Van den Berg A. V. (1998). Heading detection using motion templates and eye velocity gain fields. Vision Res. 38, 2155–2179 9797976 10.1016/s0042-6989(97)00428-8

[B9] Benjamini Y. Hochberg Y. (1995). Controlling the false discovery rate: a practical and powerful approach to multiple testing. J. R. Stat. Soc. Ser. B 57, 289–300

[B10] Bennett P. J. Sekuler R. Sekuler A. B. (2007). The effects of aging on motion detection and direction identification. Vision Res. 47, 799–809 10.1016/j.visres.2007.01.001 17289106

[B11] Berard F. Fung J. McFadyen B. J. Lamontagne A. (2009). Aging affects the ability to use optic flow in the control of heading during locomotion. Exp. Brain Res. 194, 183–190 10.1007/s00221-008-1685-1 19139863

[B12] Beusmans J. M. (1998). Perceived object shape affects the perceived direction of self-movement. Perception 27, 1079–1085 10.1068/p271079 10341937

[B13] Billino J. Bremmer F. Gegenfurtner K. R. (2008). Differential aging of motion processing mechanisms: evidence against general perceptual decline. Vision Res. 48, 1254–1261 10.1016/j.visres.2008.02.014 18396307

[B14] Bremmer F. Duhamel J. R. Ben Hamed S. Graf W. (2002a). Heading encoding in the macaque ventral intraparietal area (VIP). Eur. J. Neurosci. 16, 1554–1568 10.1046/j.1460-9568.2002.02207.x 12405970

[B15] Bremmer F. Klam F. Duhamel J. R. Ben H. S. Graf W. (2002b). Visual-vestibular interactive responses in the macaque ventral intraparietal area (VIP). Eur. J. Neurosci. 16, 1569–1586 10.1046/j.1460-9568.2002.02206.x 12405971

[B16] Bremmer F. Krekelberg B. (2003). Seeing and acting at the same time: challenges for brain (and) research. Neuron 38, 367–370 10.1016/S0896-6273(03)00236-8 12741984

[B17] Bremmer F. Kubischik M. Pekel M. Hoffmann K. P. Lappe M. (2010). Visual selectivity for heading in monkey area MST. Exp. Brain Res. 200, 51–60 10.1007/s00221-009-1990-3 19727690

[B18] Bremmer F. Lappe M. (1999). The use of optical velocities for distance discrimination and reproduction during visually simulated self motion. Exp. Brain Res. 127, 33–42 10.1007/s002210050771 10424412

[B19] Bremmer F. Schlack A. Shah N. J. Zafiris O. Kubischik M. Hoffmann K. (2001). Polymodal motion processing in posterior parietal and premotor cortex: a human fMRI study strongly implies equivalencies between humans and monkeys. Neuron 29, 287–296 10.1016/S0896-6273(01)00198-2 11182099

[B21] Britten K. H. (2008). Mechanisms of self motion perception. Annu. Rev. Neurosci. 31, 389–410 10.1146/annurev.neuro.29.051605.112953 18558861

[B22] Britten K. H. van Wezel R. J. (1998). Electrical microstimulation of cortical area MST biases heading perception in monkeys. Nat. Neurosci. 1, 59–63 10195110 10.1038/259

[B23] Browning N. A. Grossberg S. Mingolla E. (2009). A neural model of how the brain computes heading from optic flow in realistic scenes. Cogn. Psychol. 59, 320–356 10.1016/j.cogpsych.2009.07.002 19716125

[B24] Callisaya M. L. Blizzard L. Schmidt M. D. McGinley J. L. Lord S. R. Srikanth V. K. (2009). A population-based study of sensorimotor factors affecting gait in older people. Ageing 38, 290–295 10.1093/ageing/afp017 19264860

[B25] Carrozzo M. Laquaniti F. (1998). Virtual reality: a tutorial. Electroencephalogr. Clin. Neurophysiol. 109, 1–9 11003058 10.1016/s0924-980x(97)00086-6

[B25a] Carter J. H. (1982). The effects of aging upon selected visual functions: color vision, glare sensitivity, field of vision, and accommodation, in Aging and Human Visual Function, eds Sekuler R. Kline D. Dismukes K. (New York, NY: Alan R. Liss Inc), 121–130

[B26] Chen A. DeAngelis G. C. Angelaki D. E. (2011a). A comparison of vestibular spatiotemporal tuning in macaque parietoinsular vestibular cortex, ventral intraparietal area, and medial superior temporal area. J. Neurosci. 31, 3082–3094 10.1523/JNEUROSCI.4476-10.2011 21414929 PMC3062513

[B27] Chen A. DeAngelis G. C. Angelaki D. E. (2011b). Representation of vestibular and visual cues to self-motion in ventral intraparietal cortex. J. Neurosci. 31, 12036–12052 10.1523/JNEUROSCI.0395-11.2011 21849564 PMC3169295

[B28] Chou Y. H. Wagenaar R. C. Saltzman E. Giphart J. E. Young D. Davidsdottir R. (2009). Effects of optic flow speed and lateral flow asymmetry on locomotion in younger and older adults: a virtual reality study. J. Gerontol. B Psychol. Sci. Soc. Sci. 64, 222–231 10.1093/geronb/gbp003 19276239 PMC2655160

[B29] Crane B. T. (2012). Direction specific biases in human visual and vestibular heading perception. PLoS ONE 7:e51383 10.1371/journal.pone.0051383 23236490 PMC3517556

[B30] Crane B. T. (2014). Human visual and vestibular heading perception in the vertical planes. J. Assoc. Res. Otolaryngol. 15, 87–102 10.1007/s10162-013-0423-y 24249574 PMC3901863

[B31] Cuturi L. F. Macneilage P. R. (2013). Systematic biases in human heading estimation. PLoS ONE 8:e56862 10.1371/journal.pone.0056862 23457631 PMC3574054

[B41] d'Avossa G. Kersten D. (1996). Evidence in human subjects for independent coding of azimuth and elevation for direction of heading from optic flow. Vision Res. 36, 2915–2924 8917793 10.1016/0042-6989(96)00010-7

[B32] Duffy C. J. (2009). Visual motion processing in aging and Alzheimer's disease: neuronal mechanisms and behavior from monkeys to man. Ann. N.Y. Acad. Sci. 1170, 736–744 10.1111/j.1749-6632.2009.04021.x 19686221

[B33] Duffy C. J. Wurtz R. H. (1991). Sensitivity of MST neurons to optic flow stimuli. I. A continuum of response selectivity to large-field stimuli. J. Neurophysiol. 65, 1329–1345 1875243 10.1152/jn.1991.65.6.1329

[B34] Ellemberg D. Lewis T. L. Liu C. H. (1999). Development of spatial and temporal vision during childhood. Vision Res. 39, 2325–2333 10.1016/S0042-6989(98)00280-6 10367054

[B35] Elliott D. Whitaker D. MacVeigh D. (1990). Neural contribution to spatiotemporal contrast sensitivity decline in healthy ageing eyes. Vision Res. 30, 541–547 10.1016/0042-6989(90)90066-T 2339508

[B35a] Falkenberg H. K. Bex P. J. (2007). Contextual modulation of the motion aftereffect. J. Exp. Psychol. Hum. Percept. Perform. 33, 257–270 10.1037/0096-1523.33.2.257 17469967

[B36] Fetsch C. R. Turner A. H. DeAngelis G. C. Angelaki D. E. (2009). Dynamic reweighting of visual and vestibular cues during self-motion perception. J. Neurosci. 9 29, 15601–15612 10.1523/JNEUROSCI.2574-09.2009 20007484 PMC2824339

[B37] Frenz H. Bremmer F. Lappe M. (2003). Discrimination of travel distance from situated optic flow. Vision Res. 43, 2173–2183 10.1016/S0042-6989(03)00337-7 12855252

[B38] Fukushima K. (2008). Extraction of visual motion and optic flow. Neural Netw. 21, 774–785 10.1016/j.neunet.2007.12.049 18280109

[B39] Gibson J. J. (1950). The Perception of the Visual World. Boston, MA: Houghton Mifflin 10.2307/1419017

[B40] Gilmore G. C. Wenk H. E. Naylor L. A. Stuve T. A. (1992). Motion perception and aging. Psychol. Aging 7, 654–660 10.1037/0882-7974.7.4.654 1466834

[B42] Grigo A. Lappe M. (1998). Interaction of stereo vision and optic flow processing revealed by an illusory stimulus. Vision Res. 38, 281–290 10.1016/S0042-6989(97)00123-5 9536354

[B43] Grossberg S. Mingolla E. Pack C. (1999). A neural model of motion processing and visual navigation by cortical area MST. Cereb. Cortex 9, 878–895 10601006 10.1093/cercor/9.8.878

[B44] Gu Y. Angelaki D. E. DeAngelis G. C. (2008). Neural correlates of multisensory cue integration in macaque MSTd. Nat. Neurosci. 11, 1201–1210 10.1038/nn.2191 18776893 PMC2713666

[B45] Gu Y. DeAngelis G. C. Angelaki D. E. (2007). A functional link between area MSTd and heading perception based on vestibular signals. Nat. Neurosci. 10, 1038–1047 10.1038/nn1935 17618278 PMC2430983

[B46] Gu Y. DeAngelis G. C. Angelaki D. E. (2012). Causal links between dorsal medial superior temporal area neurons and multisensory heading perception. J. Neurosci. 32, 2299–2313 10.1523/JNEUROSCI.5154-11.2012 22396405 PMC3311305

[B47] Gu Y. Fetsch C. R. Adeyemo B. DeAngelis G. C. Angelaki D. E. (2010). Decoding of MSTd population activity accounts for variations in the precision of heading perception. Neuron 66, 596–609 10.1016/j.neuron.2010.04.026 20510863 PMC2889617

[B48] Gu Y. Watkins P. V. Angelaki D. E. DeAngelis G. C. (2006). Visual and nonvisual contributions to three-dimensional heading selectivity in the medial superior temporal area. J. Neurosci. 26, 73–85 10.1523/JNEUROSCI.2356-05.2006 16399674 PMC1538979

[B49] Habak C. Faubert J. (2000). Larger effect of aging on the perception of higher-order stimuli. Vision Res. 40, 943–950 10.1016/S0042-6989(99)00235-7 10720665

[B50] Harwerth R. S. Fredenburg P. M. Smith E. L. III. (2003). Temporal integration for stereoscopic vision. Vision Res. 43, 505–517 10.1016/S0042-6989(02)00653-3 12594997

[B51] Hennig P. Egelhaaf M. (2012). Neuronal encoding of object and distance information: a model simulation study on naturalistic optic flow processing. Front. Neural Circuits 6:14 10.3389/fncir.2012.00014 22461769 PMC3309705

[B52] Hennig P. Kern R. Egelhaaf M. (2011). Binocular integration of visual information: a model study on naturalistic optic flow processing. Front. Neural Circuits 5:4 10.3389/fncir.2011.00004 21519385 PMC3078557

[B52a] Heron G. Charman W. N. Schor C. M. (2001). Age changes in the interactions between the accommodation and vergence systems. Optom. Vis. Sci. 78, 754–762 10.1097/00006324-200110000-00015 11700969

[B53] Hodges L. F. (1992). Time multiplexed stereoscopic computer graphics. IEEE Comp. Graph. App. 12, 20–30 10.1109/38.124285

[B53a] Hua T. Li X. He L. Zhou Y. Wang Y. Leventhal A. G. (2006). Functional degradation of visual cortical cells in old cats. Neurobiol. Aging 27, 155–162 10.1016/j.neurobiolaging.2004.11.012 16298251

[B54] Jäncke (2004). Neuropsychologie des Alternsin, in Enzyklopädie der Gerontologie, A. Kruse and M. Martin (Bern: Verlag Hans Huber), 207–223

[B55] Kavcic V. Vaughn W. Duffy C. J. (2011). Distinct visual motion processing impairments in aging and Alzheimer's disease. Vision Res. 51, 386–395 10.1016/j.visres.2010.12.004 21156185 PMC3061234

[B56] Knox P. C. Davidson J. H. Anderson D. (2005). Age-related changes in smooth pursuit initiation. Exp. Brain Res. 165, 1–7 10.1007/s00221-005-2265-2 16021434

[B58] Lappe M. Bremmer F. Pekel M. Thiele A. Hoffmann K. P. (1996). Optic flow processing in monkey STS: a theoretical and experimental approach. J. Neurosci. 16, 6265–6285 8815907 10.1523/JNEUROSCI.16-19-06265.1996 PMC6579186

[B59] Lappe M. Bremmer F. van den Berg A. V. (1999). Perception of self-motion from visual flow. Trends Cogn. Sci. 3, 329–336 10.1016/S1364-6613(99)01364-9 10461195

[B60] Lappe M. Duffy C. J. (1999). Optic flow illusion and single neuron behaviour reconciled by a population model. Eur. J. Neurosci. 11, 2323–2331 10383621 10.1046/j.1460-9568.1999.00649.x

[B61] Lappe M. Grigo A. (1999). How stereovision interacts with optic flow perception: neural mechanisms. Neural Netw. 12, 1325–1329 10.1016/S0893-6080(99)00061-1 12662636

[B62] Lappe M. Rauschecker J. P. (1993). A neural network for the processing of optic flow from egomotion in man and higher mammals. Neural Comp. 5, 374–391

[B63] Lappe M. Rauschecker J. P. (1994). Heading detection from optic flow. Nature 369, 712–713 10.1038/369712a0 8008064

[B64] Layton O. W. Browning N. A. (2014). A unified model of heading and path perception in primate MSTd. PLoS Comput. Biol. 10:e1003476 10.1371/journal.pcbi.1003476 24586130 PMC3930491

[B65] Lee D. N. (1980). The optic flow field: the foundation of vision. Philos. Trans. R. Soc. Lond. B Biol. Sci. 290, 169–179 6106236 10.1098/rstb.1980.0089

[B65a] Leventhal A. G. Wang Y. Pu M. Zhou Y. Ma Y. (2003). GABA and its agonists improved visual cortical function in senescent monkeys. Science 300, 812–815 10.1126/science.1082874 12730605

[B66] Lipton L. (1997). Creating stereoscopic software, in StereoGraphics Developers' Handbook, (San Rafael, CA: StereoGraphics Corporation).

[B67] Mapstone M. Logan D. Duffy C. J. (2006). Cue integration for the perception and control of self-movement in ageing and Alzheimer's disease. Brain 129, 2931–2944 10.1093/brain/awl201 17071922

[B68] Mestre D. Blin O. Serratrice G. Pailhous J. (1990). Spatiotemporal contrast sensitivity differs in normal aging and Parkinson's disease. Neurology 40, 1710–1714 10.1212/WNL.40.11.1710 2234426

[B70] Munoz D. P. Broughton J. R. Goldring J. E. Armstrong I. T. (1998). Age-related performance of human subjects on saccadic eye movement tasks. Exp. Brain Res. 121, 391–400 10.1007/s002210050473 9746145

[B71] Newsome W. T. Pare E. B. (1988). A selective impairment of motion perception following lesions of the middle temporal visual area (MT). J. Neurosci. 8, 2201–2211 3385495 10.1523/JNEUROSCI.08-06-02201.1988 PMC6569328

[B72] O'Brien H. L. Tetewsky S. J. Avery L. M. Cushman L. A. Makous W. Duffy C. J. (2001). Visual mechanisms of spatial disorientation in Alzheimer's Disease. Cereb. Cortex 11, 1083–1092 10.1093/cercor/11.11.1083 11590117

[B73] Owsley C. (2011). Aging and vision. Vision Res. 51, 1610–1022 10.1016/j.visres.2010.10.020 20974168 PMC3049199

[B74] Owsley C. Sekuler R. Siemsen D. (1983). Contrast sensitivity throughout adulthood. Vision Res. 23, 689–699 10.1016/0042-6989(83)90210-9 6613011

[B75] Pakkenberg B. Gunderson H. J. (1997). Neocortical neuron number in humans: effect of sex and age. J. Comp. Neurol. 384, 312–320 9215725

[B76] Palmisano S. (1996). Perceiving self-motion in depth: the role of stereoscopic motion and changing-size cues. Percept. Psychophys. 58, 1168–1176 10.3758/BF03207550 8961828

[B77] Perrone J. A. Stone L. S. (1994). A model of self-motion estimation within primate extrastriate visual cortex. Vision Res. 34, 2917–2938 7975326 10.1016/0042-6989(94)90060-4

[B78] Prokop T. Schubert M. Berger W. (1997). Visual influence on human locomotion. Exp. Brain Res. 114, 63–70 10.1007/PL00005624 9125452

[B78a] Rambold H. Neumann G. Sander T. Helmchen C. (2006). Age-related changes of vergence under natural viewing conditions. Neurobiol. Aging 27, 163–172 10.1016/j.neurobiolaging.2005.01.002 16243410

[B79] Raz N. Lindenberger U. Rodrigue K. M. Kennedy K. M. Head D. Williamson A. (2005). Regional brain changes in aging healthy adults: general trends, individual differences and modifiers. Cereb. Cortex 15, 1676–1689 10.1093/cercor/bhi044 15703252

[B80] Roditi R. E. Crane B. T. (2012). Directional asymmetries and age effects in human self-motion perception. J. Assoc. Res. Otolaryngol. 13, 381–401 10.1007/s10162-012-0318-3 22402987 PMC3346890

[B82] Roudaia E. Bennett P. J. Sekuler A. B. Pilz K. S. (2010). Spatiotemporal properties of apparent motion perception and aging. J. Vis. 5, 1–15 10.1167/10.14.5 21131565

[B83] Roy J. P. Komatsu H. Wurtz K. H. (1992). Disparity sensitivity of neurons in monkey extrastriate area MST. J. Neurosci. 12, 2478–2492 1613542 10.1523/JNEUROSCI.12-07-02478.1992 PMC6575856

[B84] Royden C. S. Banks M. S. Crowell J. A. (1992). The perception of heading during eye movements. Nature 360, 583–585 10.1038/360583a0 1461280

[B84a] Saito H. Yukie M. Tanaka K. Hikosaka K. Fukaka Y. Iwa E. (1986). Integration of direction signals of image motion in the superior temporal sulcus of the macaque monkey. J. Neurosci. 6, 145–157 3944616 10.1523/JNEUROSCI.06-01-00145.1986 PMC6568620

[B85] Saunders J. A. Niehorster D. C. (2010). A Bayesian model for estimating observer translation and rotation from optic flow and extra-retinal input. J. Vis. 10:7 10.1167/10.10.7 20884472

[B86] Sloane M. E. Owsley C. Jackson C. A. (1988). Aging and luminance-adaptation effects on spatial contrast sensitivity. J. Opt. Soc. Am. A. 5, 2181–2190 10.1364/JOSAA.5.002181 3230488

[B87] Snowden R. J. Kavanagh E. (2006). Motion perception in the ageing visual system: minimum motion, motion coherence, and speed discrimination thresholds. Perception 35, 9–24 10.1068/p5399 16491704

[B88] Telford L. Howard I. P. (1996). Role of optical flow field asymmetry in the perception of heading during linear motion. Percept. Psychophys. 58, 283–288 8838170 10.3758/bf03211881

[B88a] te Pas S. F. Kappers A. M. L. Koernderink J. J. (1998). Locating the singular point in first order optical flow fields. J. Exp. Psychol. Hum. Percept. Perform. 24, 1415–1430 10.1037/0096-1523.24.5.1415

[B89] Tulunay-Keesey U. VerHoeve J. N. Terkla-McGrane C. (1988). Threshold and suprathreshold spatiotemporal response throughout adulthood. J. Opt. Soc. Am. A. 5, 2191–2200 10.1364/JOSAA.5.002191 3230489

[B90] Upadhyay U. D. Page W. K. Duffy C. J. (2000). MST responses to pursuit across optic flow with motion parallax. J. Neurophysiol. 84, 818–826 10938308 10.1152/jn.2000.84.2.818

[B91] van den Berg A. V. Brenner E. (1994). Why two eyes are better than one for judgements of heading. Nature 371, 700–702 10.1038/371700a0 7935814

[B91a] Vandenbossche J. Coomans D. Homblé K. Deroost N. (2014). The effect of cognitive aging on implicit sequence learning and dual tasking. Front Psychol. 5:154 10.3389/fpsyg.2014.00154 24578697 PMC3936304

[B92] von Hopffgarten A. Bremmer F. (2010). Self-motion reproduction can be affected by associated auditory cues. Seeing Perceiving 24, 203–222 10.1163/187847511X571005 21864463

[B93] Wall M. B. Smith A. T. (2008). The representation of egomotion in the human brain. Curr. Biol. 18, 191–194 10.1016/j.cub.2007.12.053 18221876

[B95] Warren W. H. Jr. Blackwell A. W. Morris M. W. (1989). Age differences in perceiving the direction of self-motion from optical flow. J. Gerontol. 44, 147–153 10.1093/geronj/44.5.P147 2768773

[B96] Warren W. H. Jr. Morris M. W. Kalish M. (1988). Perception of translational heading from optical flow. J. Exp. Psychol. Hum. Percept. Perform. 14, 646–660 10.1037/0096-1523.14.4.646 2974874

[B96a] Yu S. Wang Y. Li X. Zhou Y. Leventhal A. G. (2006). Functional degradation of extrastriate visual cortex in senescent rhesus monkeys. Neuroscience 140, 1023–1029 10.1016/j.neuroscience.2006.01.015 16678974

[B97] Zhang T. Britten K. H. (2011). Parietal area VIP causally influences heading perception during pursuit eye movements. J. Neurosci. 31, 2569–2575 10.1523/JNEUROSCI.5520-10.2011 21325524 PMC3084547

