# Supplementary material for: Self-motion perception in the elderly
Source: Front Hum Neurosci. 2014 Sep 15;8:681. doi: 10.3389/fnhum.2014.00681 (PMC4163979; doi:10.3389/fnhum.2014.00681)

Supplementary Material

Supplementary Figure 1: The panel shows the localization error as function of presentation time in the display. Red symbols show data from the test group, black symbols those from the control group.

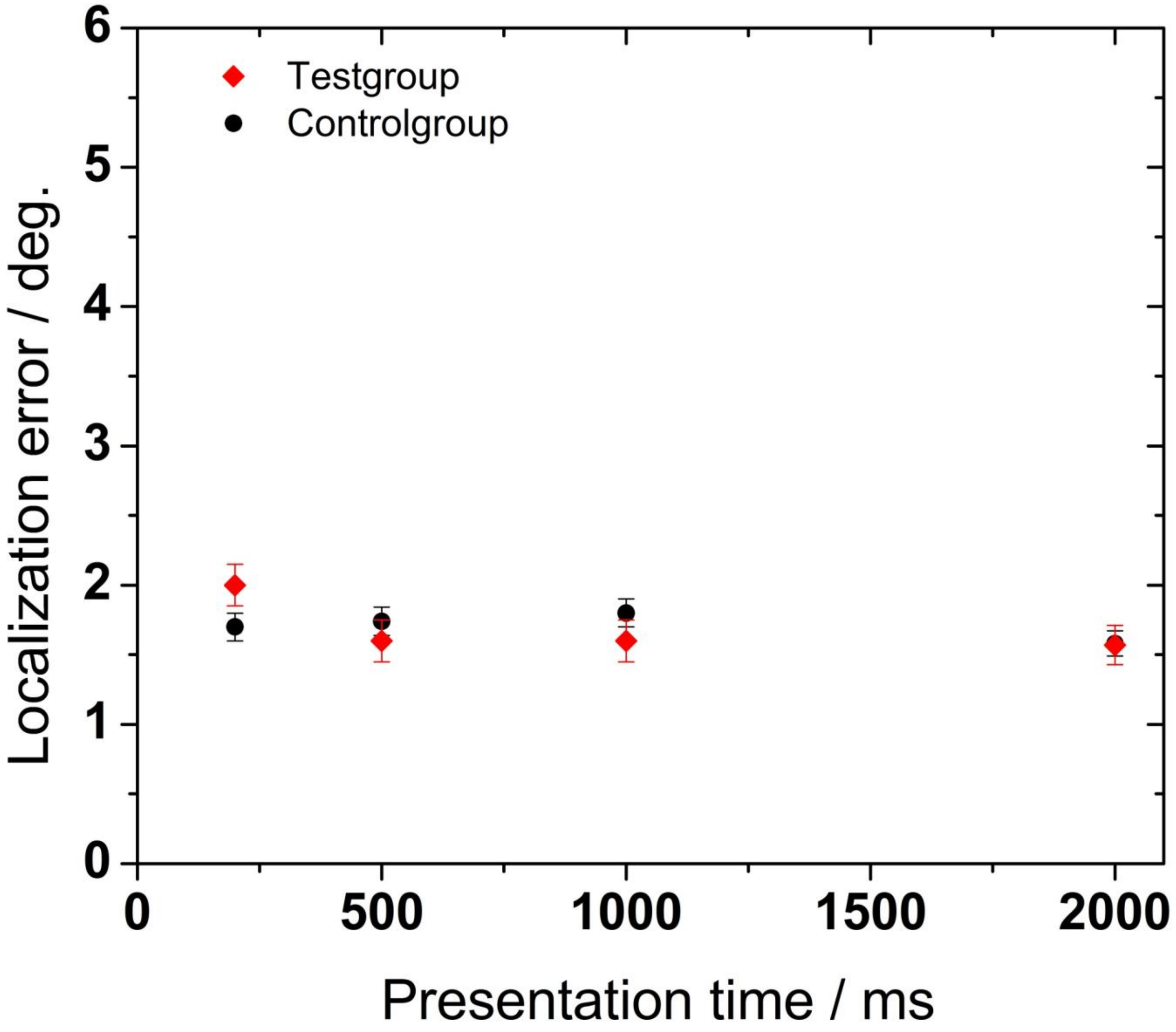

Supplement: Supplementary file 1 [file Image1.PDF]
